# Research Progress on the Mechanism of Salt Tolerance in Maize: A Classic Field That Needs New Efforts

**DOI:** 10.3390/plants12122356

**Published:** 2023-06-18

**Authors:** Jiawei Li, Qinglin Zhu, Fuchao Jiao, Zhenwei Yan, Haiyan Zhang, Yumei Zhang, Zhaohua Ding, Chunhua Mu, Xia Liu, Yan Li, Jingtang Chen, Ming Wang

**Affiliations:** 1 College of Agronomy, Qingdao Agricultural University, Qingdao 266109, China; 2 Dryland-Technology Key Laboratory of Shandong Province, Qingdao Agricultural University, Qingdao 266109, China; 3 Shandong Academy of Agricultural Science, Jinan 250100, Chinamaizesd@163.com (C.M.);

**Keywords:** maize, salt stress, osmolyte, antioxidant enzymes, reactive oxygen species

## Abstract

Maize is the most important cereal crop globally. However, in recent years, maize production faced numerous challenges from environmental factors due to the changing climate. Salt stress is among the major environmental factors that negatively impact crop productivity worldwide. To cope with salt stress, plants developed various strategies, such as producing osmolytes, increasing antioxidant enzyme activity, maintaining reactive oxygen species homeostasis, and regulating ion transport. This review provides an overview of the intricate relationships between salt stress and several plant defense mechanisms, including osmolytes, antioxidant enzymes, reactive oxygen species, plant hormones, and ions (Na^+^, K^+^, Cl^−^), which are critical for salt tolerance in maize. It addresses the regulatory strategies and key factors involved in salt tolerance, aiming to foster a comprehensive understanding of the salt tolerance regulatory networks in maize. These new insights will also pave the way for further investigations into the significance of these regulations in elucidating how maize coordinates its defense system to resist salt stress.

## 1. Introduction

Maize is one of the major crops worldwide. It is believed that it was domesticated 7000 years ago from a wild grass in Mexico. Maize can be processed into various products, including animal feed, beverages, starch, biofuel, glue, etc. [1]. In recent years, affected by global climate change, the salinization of some cultivated land became increasingly serious, which became an important negative environmental factor leading to the reduction in maize.

Salinization is a major abiotic stress around the world. Salt stress disrupts homeostasis in water potential, reactive oxygen species (ROS) level, and ion distribution [2]. In plants, salt tolerance is an important trait that allows them to grow in saline soils or be irrigated with brackish water. Therefore, plants developed many physiological and biochemical strategies to cope with salt stress, such as improving the activity of the antioxidant enzyme system, eliminating the damage of free radicals to the plant body, changing the contents of various phytohormones, ion selective absorption, ion regionalization, salt rejection, and synthesis of osmoregulatory substances [2,3,4]. After salt stress signals are transmitted to the cells, various downstream signals are activated, which can cause a series of phosphorylation cascade reactions to adapt to salt stress. Stromal closing, osmolytes, antioxidant enzymes, Na^+^/H^+^ antiporters, phytohormones, and ion transport and accumulation can be activated in response to salt stress [5]. These mechanisms are regulated by a complex network of genes and signaling pathways under salt treatment.

To study plant salt resistance, it is crucial to discover the adaptive mechanisms that plants employ to combat salt stress. Previous research on salt tolerance identified numerous resistance genes and molecular pathways that are involved in this process. Systematically analyzing the molecular genetic basis of salt tolerance in maize can have significant theoretical and practical implications, including deepening our understanding of maize’s potential for salt tolerance, developing salt-tolerant corn varieties, and enhancing maize’s ability to adapt to salt environments. Genetic engineering could be used to mitigate the effects of salt stress, and understanding the regulatory network of salt tolerance in maize can aid in achieving this goal. This review focuses on the relationship between osmolytes, antioxidant enzymes, phytohormones, ions, and salt tolerance in maize. We also raise some important questions for future studies in the conclusion section.

## 2. Osmolyte and Its Related Signaling Pathway

**Figure 1:**
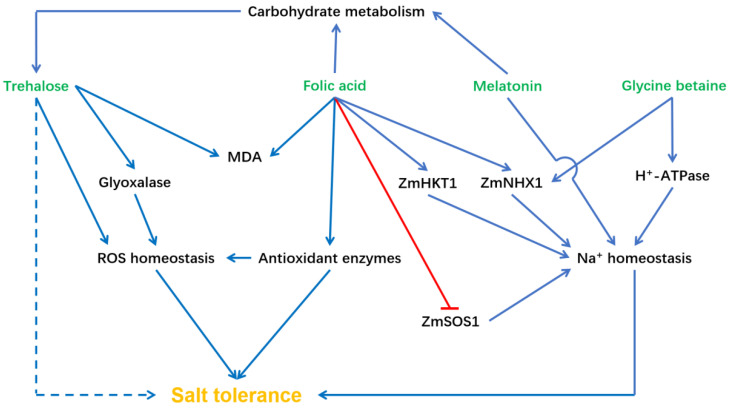
The relationship between osmolyte and salt tolerance in maize. The blue arrow means stimulation or positive effect, the red line means inhibitory effect. MDA, malondialdehyde; SOS1, salt overly sensitive 1; HKT1, high-affinity potassium transporter protein 1; and NHX1, NA^+^/H^+^ exchanger 1.

Osmolytes are low molecular weight organic compounds that are involved in maintaining the integrity of plant cells by affecting the melting point, viscosity, and ionic strength of the biological fluids [6]. Osmolytes also play an important role in combating osmotic stress caused by various environmental stimuli. Therefore, the synthesis and accumulation of osmolytes act as key regulators of combating osmotic and oxidative stress, such as salinity, cold, heat, temperature, wounding, and drought [7,8]. Trehalose is a sugar that consists of two molecules of glucose. In plants, trehalose acts as a protectant against various abiotic stresses, including heat, drought, and salt [9]. Rohman et al. (2019) showed that trehalose treatments increased root development and shoot length of maize seedlings under salt stress, with stimulated ROS levels, lipoxygenase (LOX) activity, malondialdehyde (MDA) content, glyoxalase activities, and methylglyoxal (MG) [10]. Further experiments indicated that trehalose decreased Na^+^/K^+^, ROS, MDA, and MG through influencing antioxidant enzymes and glyoxalase activities [10]. Another osmolyte, glycine betaine, can also enhance plant salt resistance [11]. Zhu et al. (2022) reported that glycine betaine can improve the growth of NaCl-treated maize seedlings by regulating cellular Na^+^ homeostasis by enhancing the transcription of the Na^+^/H^+^ antiporter gene (ZmNHX1) and H^+^-ATPase genes (ZmMHA2 and ZmMHA4) [12]. In addition, treatment of glycine betaine maintained the chlorophyll fluorescence stability, such as Fv/Fm, FPSII, and FNPQ, and activated antioxidant enzymes to mitigate salt-related growth inhibition. In addition, glycine betaine increased Na^+^ efflux in leaf protoplasts and decreased the Na^+^/K^+^ ratio primarily by increasing Na^+^ efflux from maize roots and reducing the cellular allocation of Na^+^. In maize seedlings, under salt stress, folic acid (a B vitamin) treatment can significantly improve shoot fresh weight, chlorophyll, and carotenoids, with an enhancement of antioxidant enzyme activities, cell membrane stability, and the content of relative water, free amino acids, proline, soluble sugars, K^+^, and Ca^2+^. In contrast, folic acid decreased the content of H_2_O_2_, MDA, MG, and the Na^+^/K^+^ ratio. Moreover, folic acid treatment can enhance the K^+^/Na^+^ selectivity and the performance of photosynthesis with an increased ZmHKT1 (high-affinity potassium transporter protein) transcription level and D2 protein (the major core protein of photosystem II) and a decreased expression level of ZmSOS1 and ZmNHX1 (Figure 1) [13]. 

Melatonin is a pleiotropic and functional molecule in plants. Under stress, melatonin can regulate plant development by cooperating with plant hormones and molecules, thereby influencing cell metabolism [14]. Ren et al. (2020) reported that melatonin can alleviate the salt-induced damage in maize [15]. The leaf area, biomass, antioxidant activities, and photosynthesis were higher in maize treated with melatonin compared to plants without melatonin under NaCl treatment. Melatonin treatment in maize resulted in elevated levels of osmolytes, such as sucrose and fructose. Additionally, melatonin-treated maize exhibited reduced Na^+^ content and increased K^+^/Na^+^ ratio in the shoots under salt stress (Figure 1). Muhammad et al. (2022) revealed that the application of melatonin reduced the levels of hydrogen peroxide (H_2_O_2_), superoxide anion (O_2_^−^), MDA, and electrolyte leakage under salt stress [16]. The findings led to the conclusion that melatonin mitigates oxidative damage by enhancing salt stress tolerance, regulating the antioxidant enzymes, and increasing leaf chlorophyll when compared to the control (Figure 1). Histidine is a crucial molecule that regulates ROS reduction and salt stress tolerance. Ji et al. (2022) conducted experiments to investigate the regulatory effects of histidine on the root system of maize under salt stress [17]. Their findings showed that histidine can alleviate the negative impact of salt stress on maize root growth. Treatment with histidine led to the accumulation of superoxide anion radicals, hydrogen peroxide, and MDA, while the levels of nitrate, nitrogen, and ammonium nitrogen were significantly reduced. Furthermore, the activities of SOD, POD, catalase, and nitrate metabolism-related enzymes (such as glutamine synthetase and glutamate synthase) were significantly enhanced (Figure 1). Transcriptome analysis identified that phytohormone signaling, phenylpropanoid biosynthesis, glycolysis, and nitrogen metabolism could participate in histidine-mediated salt tolerance. Polyamine (PA) is also associated with plant development and responses to various stresses. Gémes et al. (2017) showed that young transgenic tobacco with enhanced/reduced apoplastic PAO activity (S-ZmPAO/AS-ZmPAO, respectively) exhibited increased tolerance to short-term salt stress [18]. Further analysis showed that the AS-ZmPAO tobacco contained higher Ca^2+^ under salt stress, showing a reduced chlorophyll content, leaf area, and biomass under salt stress.

The accumulation of osmolytes, such as proline, soluble sugar, myo-inositol, and amino acids, was documented as a primary participant in environmental stimuli resistance. The capacity to accumulate osmolytes was correlated with salt stress tolerance in several plant species. In plants, overexpressing osmolyte production-related genes showed enhanced salt stress tolerance. All these studies show that osmolytes are prevalent for salt tolerance, and extensive investigations are required to obtain more information about osmolytes and salt stress, such as information on the relationship between osmolytes and ROS homeostasis, the key genes in osmolyte metabolism, and the kind and number of salt tolerance-related osmolytes. 

## 3. Antioxidant Enzymes and Reactive Oxygen Species

Reactive oxygen species are highly reactive chemicals formed from diatomic oxygen. Salt stress can induce ROS production, which is one of the major constraints limiting plant regulators. In plants, ROS can cause oxidative damage. Disbalance of ROS levels can result in disturbance to plant ionic homeostasis, thus affecting cell metabolism. In the process of plant growth and development, a large amount of salt stress caused by ROS accumulation in plant cells will lead to oxidative damage to chlorophyll, membranes, proteins, and nucleic acids, thus damaging normal physiological metabolism [19]. In order to avoid the accumulation of ROS, the activity of antioxidant enzyme systems in plants with strong salt resistance increased under salt stress, which can eliminate excessive ROS. Salt stress can induce the expression or the activities of some antioxidant enzymes, such as SOD, APX, POD, catalase (CAT), and phospholipid dehydrogenase glutathione peroxidase (PHGPX) synthesis [20,21,22]. Based on previous studies, the improvement of the activity of antioxidant enzymes and glutathione reductase (GR) has an important contribution to the salt resistance ability of plants. Some plants that overexpress antioxidant enzyme genes also prove that antioxidant enzymes play an important role in salt stress resistance [23,24,25,26]. In maize, the application of ROS resulted in an increasing in leaf water potential, endogenous ROS content, abscisic acid (ABA) concentration, as well as metabolite levels such as soluble sugars, proline, and polyamines. Simultaneously, it led to a decrease in lipid peroxidation and stomatal conductance [27]. In line with this, under NaCl stress, Wang et al. (2022) found that the activities of SOD, POD, CAT, APX, GR, MDHAR, and DHAR decreased, with increased content of ROS level, proline, soluble protein, soluble sugar, and MDA [28]. Jiang et al. (2017) showed selenium (Se) application increased the SOD and APX activities and the expression level of ZmMPK5, ZmMPK7, and ZmCPK11 under salt stress, with an enhanced net photosynthetic rate [29]. Se treatment also increased K^+^ in the shoots while decreasing Na^+^ in the roots, indicating that Se treatment induced the expression of ZmNHX1, a Na^+^ compartmentalization-related gene. Wang et al. (2020) showed that salt stress suppresses the photosynthetic process and induces the excessive production of ROS [30]. Salinity resulted in increased activities of antioxidant enzymes in two maize genotypes, irrespective of arbuscular mycorrhizal inoculation. However, it decreased the content of glutathione and ascorbate (non-enzymatic antioxidants). Recently, Wei et al. (2022) found that the Na^+^ concentration, Na^+^/K^+^ ratio, the contents of antioxidant enzymes and proline in maize increased under salt stress, while the contents of H_2_O_2_ and O_2_^−^ were lower [31]. Moreover, under NaCl treatment, the ROS-related gene ZmSRG7 was significantly expressed in both roots and leaves, and ZmSRG7 overexpressing mutant showed increased germination rate and root length under salt stress. Wang et al. (2022) demonstrated that the ZmBZ1, which encodes an anthocyanin 3-O-glucosyltransferase in maize, contributes to stress tolerance, especially salinity [32]. Subsequent investigations demonstrated that ZmBZ1 plays a role in scavenging ROS through anthocyanin accumulation, thereby enhancing salt stress tolerance. Liu et al. (2022) identified a salt tolerance-related nuclear pore complex (ZmNUP58). Under salt stress, overexpression of *ZmNUP58* in maize significantly enhanced both chlorophyll content and antioxidant enzymes activities [33]. RNA-seq analysis revealed that *ZmNUP58* can regulate the expression of genes involved in phytohormone synthesis and signaling, sugar metabolism, aquaporins biosynthesis, and the antioxidant enzyme system.

Salt stress enhances the production and accumulation of ROS, and causes oxidative stress. Although the research about the tolerance strategies, such as the antioxidant system and osmoregulation, significantly added to the current knowledge, more studies are needed on their relevance to complex ROS scavenging pathways. Use of the transgenic technique in the improvement of regulatory pathways in maize linked to ROS homeostasis and upregulation of the antioxidant system need to be taken as an important target.

## 4. Phytohormones

**Figure 2:**
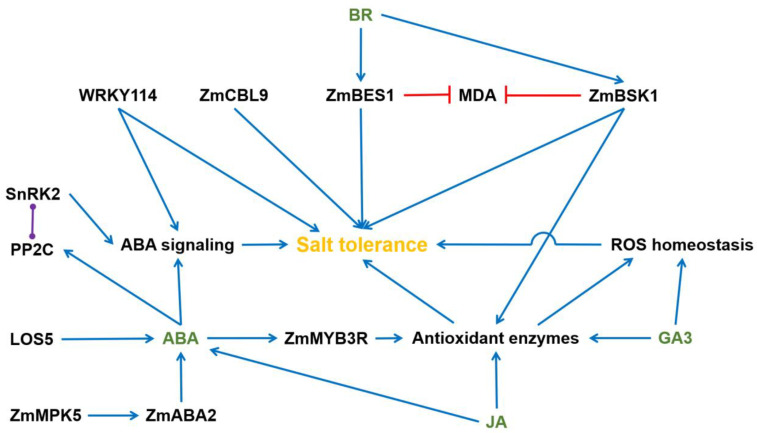
The regulatory network of plant hormones regulating salt tolerance in maize. The blue arrow means stimulation or positive effect, the red line means inhibitory effect, the violet line means protein interaction. BR, brassinosteroid; GA3, gibberellic acid; JA, jasmonic acid; ABA, abscisic acid; CBL9, calcineurin B-like proteins 9; BES1, BRI1-EMS suppressor 1; BSK1, brassinosteroid-signaling kinase 1; MPK5, MAP KINASE 5; LOS5, molybdenum cofactor sulphurase; PP2C, protein phosphatase 2C; and SnRK2, SNF1-related protein kinase.

Phytohormones are chemical compounds synthesized by plants to regulate various aspects of their growth and development. These small molecules are derived from secondary metabolism and play a crucial role in facilitating plant adaptation to environmental stimuli [34]. Normal growth and development of plants are hindered by the halophytic environment, but plants can maintain their normal development by changing the content of various hormones in their bodies under salt stress [35]. Under high salt stress, the content of ABA and cytokinin (CK) in plants increased [36,37]. ABA is a 15-C weak acid that was first identified in the 1960s. It acts as a growth inhibitor, and was shown to influence many aspects of plant growth and the development processes, such as embryo maturation, cell division and elongation, seed dormancy and germination, floral induction, and environmental stress responses [38]. ABA can induce adaptive responses of plants to salt stress, chilling injury, and osmotic stress. The ABA content in many plants increased significantly under salt stress, but the degree of increase was different in different organs. For example, under the same salt stress, ABA content in maize roots was much higher than that in leaves [39]. WRKY transcription factors are involved in various important processes in plants, including growth, sugar metabolism, defense, and stress responses. Overexpressing WRKY114 in rice led to reduced salt stress tolerance and diminished sensitivity to abscisic acid (ABA) by effecting the expression of stress- and ABA-related genes. [40]. Cai et al. (2017) cloned and characterized a new WRKY transcription factor, ZmWRKY17, from maize. Their research found that when *ZmWRKY17* was overexpressed, it resulted in increased sensitivity to salt stress while reducing sensitivity to ABA. This effect was achieved by regulating the expression of various ABA- and stress-responsive genes [41]. Furthermore, the *ZmWRKY17* overexpression mutant caused a reduced sensitivity to ABA during seed germination and early seedling growth (Figure 2). Wang et al. (2019b) showed that a salt-tolerant maize inbred line exhibited elevated activities of antioxidant enzymes (SOD, POD, APX, and CAT) and relatively lower levels of ROS accumulation under salt stress [42].

The genes involved in the ABA, ethylene, jasmonic acid (JA), and salicylic acid (SA) signal transduction pathways were upregulated in salt-tolerant maize, especially one central component, SnRK2, which positively regulates ABA signaling through various pathways [43,44]. The application of functional ABA analogue (B2) was stimulated the growth of roots. After 6 days of salt treatment, there was a significant enhancement in root growth, with a corresponding 7.6% increase in the root/shoot ratio. Additionally, the ABA level decreased by 31% compared to the control, which likely contributed to the promotion of root development. In addition to its impact on root growth, B2 demonstrated the ability to sustain higher photosynthetic capacity in maize leaves when subjected to salt stress. It achieved this by increasing the activity of antioxidant enzymes and reducing the rate of ROS generation by 16.48%. Furthermore, B2 exhibited the capacity to enhance water absorption by upregulating the expression of the aquaporin gene ZmPIP1 [45]. Chen et al. (2021) demonstrated that alternative splicing is likely to mediate early responses to salt stress during maize seed germination by using RNA-seq and SWATH-MS-based quantitative proteomics [46]. Interestingly, ABA levels in isolated embryos under NaCl treatment are unable to increase in comparison with those of the water control, indicating that the ABA elevation is an endosperm-dependent process. The calcium signaling pathway plays a crucial role in plant growth and development. It can mediate ABA-related defense mechanisms by interacting with PP2Cs (protein phosphatase 2Cs), SnRK2s (SNF1-related protein kinase 2s), and MAPK (mitogen-activated protein kinase) [47]. A genome-wide association study (GWAS) of 300 salt-treated maize accessions identified a PP2C protein that could be involved in salt tolerance through the ABA signaling pathway [48]. In line with that, He et al. (2019) showed that the majority of ZmPP2C genes exhibited a significant increase in expression levels in response to both salt stress and ABA. Notably, when ZmPP2C-A1, ZmPP2C-A2, and ZmPP2C-A6 were overexpressed, the resulting transgenic plants displayed higher germination rates following treatments with ABA and NaCl. These findings suggest that the PP2C protein likely plays a role in the response to salt stress by engaging in ABA-mediated signaling pathways (Figure 2) [49]. Calcineurin B-like proteins (CBLs) play an important role in regulating calcium signaling in response to various abiotic stresses by interacting with CIPKs (specific CBL-interacting protein kinases). Chen et al. (2011) identified 43 putative *ZmCIPK* genes in maize. Microarray data and RT-PCR assays show that 24 *ZmCIPK* genes were upregulated by salt stress [50]. Zhang et al. (2016) identified a calcineurin B-like protein, ZmCBL9, which negatively regulates the expression of genes in the ABA signaling, biosynthesis, and catabolism pathways (Figure 2) [51]. Overexpression of *ZmCBL9* enhanced resistance to ABA and salt stress. Sun et al. (2015) identified 20 nonredundant MAPK genes via a genome-wide survey in maize [52]. The transcription level of most ZmMAPKs changed significantly under salt or ABA treatments, implying that they might be involved in ABA signaling and ABA-induced antioxidant defense. In line with this, Ma et al. (2016) showed that salt stress could induce the expression of *ZmABA2* (a member of the short-chain dehydrogenase/reductase family) in maize and regulate the content of ABA [53]. Under salt stress, *ZmABA2* overexpression mutant increased the ABA level and changed seed germination and root growth. Further experiments confirmed that ZmABA2 is a direct target of ZmMPK5 and participates in ABA biosynthesis and functions [53]. The actin-depolymerizing factor (ADF) is a small class of actin-binding proteins and can be phosphorylated by a calcium-stimulated protein kinase [54]. Huang et al. (2020) demonstrated that the transcription of ADFs mainly responded to salt, drought, and ABA. LOS5, a molybdenum cofactor sulfurase, is a key regulator of ABA biosynthesis [55]. Overexpressing LOS5 in maize significantly stimulated the expression level of ZmVp14-2, ZmAO, and ZmMOCO, and increased aldehyde oxidase activities. This cascade resulted in enhanced accumulation of ABA in transgenic plants when treated with salt stress. The LOS5 overexpression mutant further stimulated the expression of ABA biosynthetic genes, promoting ABA accumulation. This, in turn, activated the expression of ion transporter genes and PIP aquaporin genes, thereby regulating root ion fluxes and water uptake. Consequently, the transgenic maize plants exhibited improved maintenance of cytosolic K^+^ and Na^+^ homeostasis, along with proper water status, under salt stress (Figure 2) [56]. Overexpressing LOS5 also induced the expression of ZmNHX1, ZmCBL4, and ZmCIPK16, which facilitated increased net Na^+^ efflux and H^+^ influx in the roots while reducing net K^+^ efflux. This mechanism played a crucial role in maintaining a high cytosolic K^+^/Na^+^ ratio in the transgenic plants when confronted with salt stress [56]. Wu et al. (2019) reported that a maize MYB transcription factor, ZmMYB3R, was induced by salt and ABA [57]. Overexpressing ZmMYB3R in Arabidopsis resulted in enhanced growth performance and higher survival rates, increased activities of CAT, POD, and SOD, and elevated sensitivity to ABA, suggesting that ZmMYB3R enhances tolerance to salt stress via an ABA-dependent pathway (Figure 2).

Jasmonates (JAs) are lipid-derived endogenous hormones that act as one of the key regulators of both developmental processes and different defense responses in plants. A previous study showed that the application of JA could alleviate the harmful effect caused by NaCl stress by improving antioxidant enzyme activities and radical scavenging capacity [58]. In maize seedlings, Ahmad et al. (2019) showed that JA plays a role in salt-inducing cell death and subsequent leaf senescence by using a JA biosynthesis mutant [59]. Furthermore, foliar stomatal observation and ion analysis indicated that JA is positively involved in regulating the movement of guard cells during salt stress. The absence of JA resulted in increased salt-related damage on the roots but reduced stress on the leaves of the seedlings. Analysis of ROS level demonstrated that the JA biosynthesis mutant exhibited lower H_2_O_2_ levels in the leaves but higher levels in the roots when subjected to salt treatment. Correspondingly, the activity of antioxidant enzymes displayed a similar pattern. Additionally, the expression levels of four crucial enzymes involved in ABA biosynthesis, namely ZEP1, NCED5, AO1, and VP10, were significantly downregulated in the shoots under salt treatment (Figure 2). Notably, endogenous JA served as a positive regulator for the transport of Na^+^ from the roots to the shoots, as evidenced by the mutant’s higher Na^+^ levels in the roots but significantly lower levels in the shoots. Moreover, JA likely acts as a positive regulator of ABA biosynthesis in leaves when subjected to salt stress. In line with this, Mir et al. (2018) examined the role of JA in improving Na_2_CO_3_-induced salt stress [60]. Maize seedlings treated with JA significantly mitigated the toxic effects of Na_2_CO_3_ by decreasing Na^+^ uptake and accumulating ROS and MDA levels (Figure 2). In addition, JA treatment also stimulated antioxidant enzyme activities and ABA content and counteracted the salt-induced proline and glutathione content. Salicylic acid (SA) is an essential plant defense hormone and participates in stress resistance [61]. In maize, Elhakem (2020) showed that SA treatment mitigated the negative impact of salt stress through an elevated level of TSS, TSP, proline, K^+^, Ca^2+^, IAA, and GA3 but decreased the Na^+^, Na^+^/K^+^ ratio, and ABA to an appreciable level [62].

Gibberellic acid (GA) is an endogenous plant growth regulator. Under salt stress, GA3 treatment can alleviate the physiological parameters that cause salt stress. GA3 application improved maize growth, reduced the H_2_O_2_ content, Na^+^ concentration, and oxidative stress, and elevated the antioxidant enzyme activities, antioxidant gene expression, and K^+^ concentration [63]. In maize leaves, GA3 treatment triggered glutathione S-transferase activities as well as hydrogen sulfide accumulation and anthocyanin content, with reduced MDA, H_2_O_2_, and O_2_^−^ content (Figure 2) [64].

Brassinosteroids are a class of polyhydroxylated steroidal phytohormones in plants and regulate a wide range of physiological processes [65]. The BRI1-EMS suppressor 1 (BES1)/brassinazole-resistant 1 (BZR1) transcription factors, which serve as essential components in the brassinosteroid signaling pathway, play crucial roles in plant growth and development. Sun et al. (2020) characterized ZmBES1 from maize. It was found that ZmBES1 is localized in the nucleus and exhibits responsiveness to both ABA and salt stress [66]. Through heterologous expression of ZmBES1, several notable effects were observed. Firstly, there was a reduction in ABA sensitivity, accompanied by facilitated shoot growth and enhanced root development. Additionally, ZmBES1 overexpression resulted in improved salt tolerance, as indicated by lower levels of MDA content and relative electrolyte leakage (Figure 2). Liu et al. (2022) reported that the maize brassinosteroid signaling kinase gene, ZmBSK1, plays a significant role in the response to salt stress [67]. The transcript level of ZmBSK1 exhibited upregulation in maize leaves, roots, and stems when exposed to NaCl treatment. Furthermore, overexpression of ZmBSK1 led to enhanced salt tolerance in maize. The ZmBSK1 overexpression mutant showed modulated expression levels of ROS-scavenging and proline biosynthesis-related genes, increased antioxidant enzyme activities, and reduced MDA content, electrolyte leakage percentage, and O_2_^−^ and H_2_O_2_ accumulation (Figure 2).

Gretchen Hagen 3 (GH3) genes play an important role in regulating auxin homeostasis by catalysing auxin conjugation and binding free indole-3-acetic acid (IAA) to amino acids [68]. Feng et al. (2015) demonstrated that the expression patterns of ZmGH3 genes respond to salt stress, indicating their potential involvement in enhancing maize tolerance to environmental stresses [69]. The polar transport of auxin relies on various protein families, including auxin influx carriers such as auxin-resistant 1/like aux 1 (AUX/LAX), efflux carriers such as pin-formed (PIN) proteins (along with PIN-like proteins), and efflux/conditional P-glycoprotein (ABCB). Under salt stress, the expression levels of most ZmPIN, ZmPILS, ZmLAX, and ZmABCB genes were observed to be induced in shoots, but reduced in roots [70].

Plant hormones, such as ABA, SA, JA, ethylene, auxin, CKs, GA, and BRs, were found to play a crucial role in mediating salt stress signals and controlling the balance between growth and stress responses. Some plant hormones regulate salt tolerance positively, whereas others play a negative role. Most of our knowledge about the relationship between plant hormones and the salt stress response comes from studies in some model plants. Compared with model plants, our knowledge about the regulatory mechanism of hormone-mediated salt stress in maize is still limited. For example, how do plant hormones regulate ROS homeostasis? What is the regulatory mechanism by which plant hormone signaling influences ion transport? Can we improve the resistance of maize to salt stress by modifying some key genes without affecting the yield? Therefore, further studies are needed to discover the role of plant hormones in salt tolerance and to identify key genes to guide maize production.

## 5. The Role of Na^+^, K^+^, and Cl^−^ in Maize Tolerance to Salt Stress

Plants need to maintain the dynamic balance required for intracellular physiological metabolism through selective absorption, efflux, and ion regionalization. The content of Na^+^, K^+^, and Cl^−^ ions, as well as their homeostasis, is important for preventing salt damage.

### 5.1. Na^+^

Sodium (Na^+^) is one of the most abundant soluble cations in salinized soil. Excessive accumulation of Na^+^ in plants can lead to Na^+^ poisoning, resulting in stunted growth and even plant death [71,72]. Therefore, maintaining Na^+^ homeostasis is crucial for plants to resist salt stress. Previous studies identified several key factors that regulate Na^+^ homeostasis in plants, including Na^+^ selective transporters that play an important role in maintaining plant Na^+^ balance [73]. In a study by Wei et al. (2022), it was found that ZmSRG7 expression level was stimulated in both roots and leaves under salt stress [31]. Maize overexpressing ZmSRG7 showed an increased germination rate and root length under salt stress, accompanied by higher Na^+^ concentration and Na^+^:K^+^ ratio. Additionally, the levels of antioxidant enzymes and proline in maize under salt stress were higher, while the levels of MDA, H_2_O_2_, and O^2−^ were lower compared to the control. Further analysis revealed that ZmSRG7 can interact with ZmDHN1, which is involved in maintaining H_2_O_2_ homeostasis. Zörb et al. (2005) identified six Na^+^/H^+^ antiporters (ZmNHX1-6) in maize [74]. The expression of ZmNHX is upregulated in roots when exposed to high NaCl concentrations, showing an organ and salt-specific pattern. Ali et al. (2021) demonstrated that salt-induced phytotoxicity increased the concentration of MDA, H_2_O_2_, Na^+^/K^+^ ratio, Na^+^ translocation (root to shoot), and its uptake [23]. The mitigation of salt stress by silicon was attributed to a decrease in Na^+^/K^+^ ratio, reduced Na^+^ uptake at the surface of maize roots, and diminished Na^+^ accumulation in plant tissues. The transport of Na^+^ in maize xylem vessels is a key physiological process for maintaining Na^+^ homeostasis under salt stress [23]. Na^+^ selective ion transporters belonging to different families synergistically contribute to Na^+^ unloading [75,76]. Zhang et al. (2018) reported that ZmHKT1, an HKT-type transporter, could be involved in maize salt tolerance [75]. The presence of a natural ZmHKT1 loss-of-function allele, which contains a retrotransposon insertion, leads to an accumulation of Na^+^ in leaves and increased sensitivity to salt. Loss of ZmHKT1 function mutant elevated Na^+^ concentration in the xylem sap, resulting in enhanced delivery of Na^+^ from the roots to the shoots. This indicates that ZmHKT1 facilitates leaf Na^+^ exclusion and promotes salt tolerance by eliminating Na^+^ from the xylem sap. Furthermore, Zhang et al. (2019) identified a novel membrane-localized Na^+^-selective transporter involved in shoot Na^+^ exclusion [76]. The presence of a natural ZmHAK4-deficient allele was associated with decreased ZmHAK4 expression and increased Na^+^ content in the shoots. ZmHAK4 was found to be predominantly expressed in the root stele and is likely responsible for retrieving Na^+^ from the xylem sap. The results also highlight that ZmHAK4 and ZmHKT1 have distinct roles in facilitating shoot Na^+^ exclusion and promoting salt tolerance.

Furthermore, Cao et al. (2020) demonstrated substantial variations in shoot Na^+^ content and saline–alkaline (NaHCO_3_) tolerance among natural maize inbred lines. They identified ZmNSA1 (Na^+^ content under saline–alkaline condition) through a genome-wide association study as a gene responsible for shoot Na^+^ variations under NaHCO_3_ conditions [77]. Knocking out ZmNSA1 promotes shoot Na^+^ homeostasis by increasing root Na^+^ efflux. Moreover, under salt conditions, Ca^2+^ binds to the EF-hand domain of ZmNSA1, triggering its degradation via the 26S proteasome. This, in turn, leads to an increase in the transcript levels of PM-H^+^-ATPases (MHA2 and MHA4), enhancing the salt overly sensitive 1 (SOS1)-mediated root Na^+^ efflux [78]. Previous studies also reported a close relationship between Ca^2+^ and Na^+^ toxicity [78,79]. Ca^2+^ can alleviate Na^+^ toxicity by regulating the SOS signaling pathway [80,81]. The SOS signaling pathway, which includes SOS3, SOS2, and SOS1, is believed to mediate cellular signaling under salt stress to maintain ion homeostasis. Among them, SOS3 encodes a Ca^2+^-binding protein that functions as the primary calcium sensor, perceiving the increase in cytosolic Ca^2+^ triggered by excess Na^+^ [82]. Genes involved in the Ca^2+^-mediated SOS signaling pathway may serve as important genetic resources for breeding salt-tolerant maize.

### 5.2. K^+^

Potassium (K^+^) is a crucial and abundant cation within plant cells, serving as an essential nutrient. Plants possess diverse transport systems dedicated to acquiring K^+^, enabling them to facilitate K^+^ uptake across a broad range of external concentrations. Additionally, these transport systems play a vital role in mediating the movement of K^+^ within the plant and facilitating its efflux into the environment [83]. Salt-tolerant maize hybrid Pioneer 32B33 and Pioneer 30Y87, which have high K^+^/Na^+^ ratios, showed a tight relationship between K^+^ content and salt tolerance [84]. Furthermore, Cao et al. (2019) revealed that ZmHKT2 is a major QTL that regulates K^+^ homeostasis in saline soils, by using a maize (W22)–teosinte recombinant inbred line (RIL) [85]. ZmHKT2 is responsible for encoding a transporter belonging to the K^+^-preferring HKT family. Its primary function is likely to reduce the K^+^ content in the shoots by actively eliminating K^+^ ions from the flowing xylem sap, which moves from the roots to the shoots. When ZmHKT2 is deficient, there is an elevation in both the xylem sap and shoot K^+^ concentrations, ultimately leading to an increase in salt tolerance. The presence of a coding sequence polymorphism in the ZmHKT2 W22 allele results in an amino acid variant known as ZmHKT2. This variant leads to an enhanced concentration of K^+^ in the xylem sap, subsequently increasing the shoot K^+^ content and conferring greater salt tolerance.

### 5.3. Cl^−^

Cl^−^ is an essential micronutrient in plants [86]. The toxicity threshold of Cl^−^ is estimated to be 15–50 and 4–7 mg per gram dry weight for Cl^−^-tolerant and sensitive species, respectively [87]. Cl^−^ regulates many physiology processes in plants, such as photosynthesis, membrane stabilization, enzyme activities, pH regulation, etc. [87]. In recent years, the role of Cl^−^ in salt tolerance attracted more attention. Geilfus et al. (2018) revealed that Cl^−^ can induce the increase in leaf tissue ABA concentrations in maize [88]. Nitrate transporter 1/peptide transporter family (NPF) 6.3 is a dual-affinity plasma membrane transport protein, and was reported to be involved in salt, cold, and low nitrogen stress [89]. A study conducted on two maize homologs of NPF6.3 (ZmNPF6.6 and ZmNPF6.4), revealed interesting functional characteristics. ZmNPF6.4 was identified as a low-affinity nitrate transporter that primarily facilitates the efflux of nitrate. However, when exposed to chloride, ZmNPF6.4 exhibited a remarkable shift in function, transitioning into a high-affinity transporter with selectivity towards chloride ions. On the other hand, ZmNPF6.6 displayed low-affinity transport activity for chloride ions, indicating its limited role in chloride transport [90]. Luo et al. (2021) performed a genome-wide association study on 348 maize inbred lines under normal and salt stress conditions, and identified 104 quantitative trait loci (QTLs) [91]. Based on functional annotations and chloride content analysis, they found that ZmCLCg (a chloride transport) had a potential role in salt tolerance. Ligaba et al. (2012) showed that ZmALMT2 has a high permeability for NO_3_^−^ and Cl^−^ [92]. Moreover, ZmALMT2 also exhibits a high permeability to organic anions such as malate and citrate, which could participate in the response to biotic and abiotic stresses [93].

Under salt stress, the abnormal Na^+^ concentration in the cytoplasm will cause ion toxicity to the physiological activities in the cells [79]. Plant cells can ensure the normal physiological activities of cells by salt rejection or regionalization of toxic ions, and maintain appropriate K^+^ and Ca^+^ concentrations, which is one of the important mechanisms for plants to adapt to salt stress [3]. K^+^ is a necessary ion and an important osmoregulation component for plant growth and development. Because the radii and hydration energies of the two ions are similar, Na^+^ has a significant competitive inhibition on K^+^ absorption. Plants under salt stress are often damaged by both Na^+^ toxicity and K^+^ deficiency, and the degree of their selection became an important factor affecting the salt resistance of plants [3,94,95]. Therefore, it is necessary for plant growth and salt tolerance to keep the K^+^ concentration in plant cytoplasm higher than a certain value. Furthermore, the activity of the Na^+^/H^+^ antiporter protein increases under salt stress, which enhances the ability to transport Na^+^. Under the energy provided by proton pump, the protein either segregates Na^+^ into vacuoles or discharges it to the outside of plants, thereby reducing the concentration of Na^+^ in cytoplasm, leaving excessive Na^+^ from metabolic sites, and alleviating its damage to enzymes and membrane systems [3,95,96]. In the future, the key regulation network and some important genes between ions and salt stress need to be studied deeply. Whether there exist new ions that are involved in maize salt stress also needs to be answered.

## 6. Conclusions and Future Prospects

Salt stress is one of the most significant environmental challenges limiting plant productivity. The initial recognition of salt stress occurs within the root system, leading to the induction of osmotic stress and subsequent limitations in water availability [97]. Salt stress induces ion toxicity due to nutrient imbalances in the cytosol. Plant cells need to undergo large changes to respond and defend against salt stress. Various studies showed that ion transport and ion content regulate salt tolerance in plants. Under salt stress, increasing the expression of genes encoding high-affinity K^+^ transport systems and K^+^ channels and expelling excessive salt from cells will improve the salt tolerance of plants [98]. Plants excrete Na^+^ and vacuole partition Na^+^ to reduce Na^+^ accumulation caused by toxicity, which is completed by the Na^+^/H^+^ reverse transporter. Since then, plant Na^+^/H^+^ antiporter proteins were reported on the plasma membrane and vacuole membrane. Na^+^/H^+^ antiporters rely on the proton-driven force generated by H-ATPase or H-ppase to transport Na^+^ and play an important role in plant salt tolerance [3,99]. Under salt stress, cells activate ion transporters and channels to reestablish ion balance and ROS homeostasis. In the ion transport process, K^+^ influx, Na^+^ exclusion, Ca^2+^ pumping, the Na^+^/K^+^ ratio, and Na^+^/H^+^ transport act as important regulators of plant salt tolerance [100]. For example, ZmHKT1, ZmHAK4, and ZmNSA1 regulate Na^+^ homeostasis in maize under salt and saline–alkali stress conditions, ZmHKT2 maintains the K^+^/Na^+^ balance, and ZmCS3, ZmUGT, and ZmCYP709B2 participate in the regulation of salt-induced osmotic stress. In plants, salt stress decreased the relative water content (RWC), chlorophyll (Chl), and carotenoid (Car) contents, membrane stability index (MSI), and K^+^ and Ca^2+^ contents and increased the rate of O_2_^−^ production, H_2_O_2_ content, thiobarbituric acid reactive substances (TBARS) (measure of lipid peroxidation), proline, glycine betaine, total soluble sugars, Na^+^, and Na^+^/K^+^ and Na^+^/Ca^2+^ ratios in both genotypes. The activities of SOD, APX, catalaseCAT, and glutathione reductase (GR) increase under salt stress [101]. Recently, research on the molecular mechanism of salt tolerance in maize made progress in many aspects, and multiple QTL genes related to salt tolerance in maize were cloned through GWAS, QTL, and other experiments. Liang et al. (2021) profiled the metabolomes of 266 maize inbred lines under control and salt stress and identified 37 metabolite biomarkers, which showed a tight relationship between salt tolerance and sugar metabolism [102]. Sugar metabolism is an important energy source in plants, which was shown to participate in salt tolerance deeply [33]. In other words, enhancing energy application could help plants resist the negative effect of salt stress. Proline was long recognized as a reliable stress marker in various plant species, and plays a vital role in mitigating the adverse effects of salt stress on plant cells [103]. The accumulation of proline in plant cells serves multiple protective functions. Firstly, proline acts as an osmoprotectant, maintaining cell turgor and protecting cellular structures from damage caused by water deficit or excessive salinity. Secondly, proline exhibits potent antioxidant properties, effectively scavenging ROS and reducing oxidative stress induced by salt stress. Additionally, proline plays a crucial role in stabilizing proteins, maintaining their structure and function under salt conditions [104,105]. Proline accumulation serves as a reliable and versatile stress marker, reflecting the adaptive responses of plants to various environmental challenges [103,105]. The understanding of proline metabolism and its role in stress tolerance can contribute to the development of innovative strategies to enhance maize productivity and sustainability under salt conditions. The identification and analysis of these genes significantly enhanced our understanding of the molecular mechanism of salt tolerance in maize. Furthermore, they provided valuable genetic resources and screening targets for the breeding of salt-tolerant maize varieties. Reverse genetics methods were also employed to identify several genes that may play a crucial role in the salt tolerance response in maize. Understanding the salt stress signaling pathway and characterizing the upstream salt stress sensors can guide the development of effective strategies to mitigate the negative impact of salt stress on crop yields and ultimately improve agricultural productivity. In particular, identifying the upstream regulators, characterizing high-resolution sensors, transporters, and channels of Na^+^ and K^+^, and discovering novel channels and pools of Ca^2+^ will be the focus of future research in this area.

Despite advancements in our understanding of salt stress tolerance in maize, the translation of this knowledge into substantial improvements in maize salt tolerance is limited. Firstly, the complexity of salt stress tolerance in maize involves the interplay of multiple genetic, physiological, and biochemical mechanisms. While individual genes and pathways were identified and characterized, the intricate regulatory networks and interactions among these components are still not fully elucidated. The lack of a comprehensive understanding of the underlying mechanisms hampers targeted genetic engineering and breeding efforts for enhanced salt tolerance. Furthermore, the genetic diversity of maize cultivars poses a challenge in transferring salt tolerance traits across different genetic backgrounds [106,107]. Therefore, efforts to develop salt-tolerant maize varieties must consider the genetic variability and adaptability of different germplasms. In conclusion, the limited progress in achieving substantial increases in maize salt tolerance can be attributed to the complexity of salt stress tolerance mechanisms, genetic diversity among maize cultivars, challenges in reproducing field conditions, and the time and resources required for practical implementation. Addressing these challenges will require a multidisciplinary approach, involving collaborations among plant scientists, breeders, and agronomists, as well as the utilization of innovative tools such as genomic selection, marker-assisted breeding, and gene editing technologies.

## Data Availability

Not applicable.
